# Formation of *Streptococcus mutans* Polymicrobial Biofilms in the Presence of *Lactobacillus plantarum* and *Candida albicans*

**DOI:** 10.1055/s-0045-1806962

**Published:** 2025-05-02

**Authors:** Indah Listiana Kriswandini, Christian Khoswanto, Muhammad Luthfi, Pinta Rahayuning Tyas, Adelheid Chrissanda Hermanto, Mohammed Ahmed Aljunaid

**Affiliations:** 1 Department of Oral Biology, Faculty of Dental Medicine, Universitas Airlangga, Surabaya, Indonesia; 2 Student of Dental Medicine Education Study Program, Faculty of Dental Medicine, Universitas Airlangga Surabaya, Indonesia; 3 Department of Dental Medicine, Faculty of Medicine, Taiz University, Taiz Yemen

**Keywords:** cariogenic biofilms, quorum sensing, *Streptococcus mutans*, *Lactobacillus plantarum*, *Candida albicans*

## Abstract

**Objective:**

Dental caries is an infectious disease that develops through biofilm. *Streptococcus mutans* is a cariogenic bacterium that can be found in dental plaque. *Streptococcus mutans* regulates biofilm formation and communicate with other microbes through a process called quorum sensing. Dental caries prevention can be achieved by inhibiting quorum sensing. This study aimed to investigate the ability of *Lactobacillus plantarum* and *Candida albicans* to inhibit the formation of *S. mutans* polymicrobial biofilms *.*

**Materials and Methods:**

This study aims to investigate the ability of biofilm formation analyzed through the crystal violet (CV) assay and bacterial metabolic activity analyzed through the methylthiazol tetrazolium (MTT) assay. The bacteria used are *S. mutans* (serotype C), *L. plantarum* (FNCC 0020), and *C. albicans* .

**Results:**

CV assay results show that in the presence of *L. plantarum* , biofilm formation in *S. mutans* decreases (9.5%). Meanwhile, the formation of *S. mutans* biofilms increased with the presence of *C. albicans* (28.8%). MTT assay results showed an increase in the metabolic activity of *S. mutans* in the presence of *L. plantarum* (20.2%) and *C. albicans* (19.4%).

**Conclusion:**

*Lactobacillus plantarum* can inhibit the formation of *S. mutans* biofilms, while *C. albicans* can increase *S. mutans* biofilms.

## Introduction

Biofilms, known as physiological stages that play an important role in the life of microorganisms, can be a pathological stage that acts as a medium for the spread of disease. 1 These biofilms can be formed, either by a single species (monospecies biofilms) or by more than two different species called polymicrobial biofilms. 2

Caries is a condition where there is demineralization of the hard tissue of the tooth and is associated with the formation of *Streptococcus mutans* biofilm. *Streptococcus mutans* is a normal flora in the oral cavity and acts as an early colonizer as a place of attachment for other microbes to form a polymicrobial biofilm. 3 4 *Streptococcus mutans* is a cariogenic bacterium that is both acidogenic (able to utilize plaque for acid production) and aciduric (able to survive under acidic conditions). This is related to dental caries progression. 5

Regulation of biofilm formation occurs through a mechanism called quorum sensing. Quorum sensing is a signaling mechanism process in microbes. This process is achieved through the production, secretion, and response of a molecule called an inducer as signaling molecules. When it has reached the threshold, there will be changes in gene expression and the nature of the bacteria within the biofilm. 6 The quorum sensing process of *S. mutans* biofilm formation is facilitated by competence stimulating peptide (CSP) via the two-component pathway (ComDE). CSP regulates the transcription of specific target genes in biofilm formation, namely, glucosyltransferase B/C/D ( *gtf B/C/D* ), fructosyltransferase ( *ftf* ), and glucan-binding protein B ( *gbpB* ) which is responsible to convert sucrose into glucose and fructose and form a glucan polymer called EPS which functions in the process of attachment and accumulation of bacteria in the formation of cariogenic biofilms. 7 8 Inhibition of the quorum sensing process in *S. mutans* biofilm formation can be considered as one of the approaches to prevent caries.

This study aimed to investigate the ability of *Lactobacillus plantarum* and *Candida albicans* to inhibit the formation of *S. mutans* polymicrobial biofilms. In dental plaque, *S. mutans* has the potential to communicate with other microbes, including *L. plantarum* and *C. albicans* . According to Wasfi et al, *L. plantarum* was proven to reduce *gtfB* and *gtfD* expression, which leads to reduction of *S. mutans* biofilm formation. 9 According to a research study by Arias et al, who tested the effect of tyrosol, which is an inducer of *C. albicans* , on single and mixed biofilms from *S. mutans, C. albicans* , and *Candida glabrata* showed that tyrosol in high concentrations can inhibit biofilms both in singles and mixed. 10 In this study, specific gene expression was not looked at, but the results of biofilm formation were seen as a result of this research *.*

## Materials and Methods

### Bacterial Strains and Growth Condition

This is a laboratory experimental research study conducted at the Research Center Laboratory of the Faculty of Dentistry, Airlangga University, Surabaya. This study was conducted between September and October 2023. The bacteria used, namely *S. mutans serotype* c and *C. albicans,* were obtained through stocks at the Research Center of the Faculty of Dentistry, Universitas Airlangga. Meanwhile, *L. plantarum* FNCC 0020 was obtained through stocks from the Center for Food and Nutrition Studies, Universitas Gadjah Mada. *Lactobacillus plantarum* was grown in de *Man, Rogosa, and Sharpe* (MRS) agar media first. Meanwhile, *C. albicans* is grown in *Sabouraud Dextrose Agar* (SDA) media. Both were then incubated at 37°C for 48 hours anaerobically. After that, *S. mutans, L. plantarum,* and *C. albicans* were grown in trypticase soy broth (TSB) media coupled with 5% sucrose, which was then incubated for 24 hours at 37°C. After 24 hours, the bacteria were compared with 0.5 McFarland solution (10 ^8^ FU/mL).

### Bacterial Cultivation

For bacterial cultivation, 150 μL of TSB media is inserted into a microplate well as a control. Then, the suspension of each bacterium was put into a microplate well of 150 μL for monospecies biofilm cultivation. For dual-species biofilm cultivation ( *S. mutans* with *L. plantarum* and *S. mutans* with *C. albicans* ), 75 μL of each bacterium was added. Furthermore, for the cultivation of polymicrobial biofilms formed by *S. mutans* with *L. plantarum* and *C. albicans* , each suspension of test bacteria was put into 50 microplate wells. All total suspensions in one well are 150 μL and six times replicated per treatment. Then, the microplate was incubated at 37°C for 24 hours anaerobically.

### Crystal Violet Dye Preparation

After incubating for 24 hours, the remaining solution was removed and the microplate was washed with 200 μL of *phosphate-buffered saline* solution three times. When washing, the microplate was patted to remove the solution that is still in the drain. The microplate was then dried upside down for 10 minutes.

### Crystal Violet Assay

Biofilm staining was carried out using 110 μL of 0.4% crystal violet solution in each well and then allowed to stand for 15 minutes. The dye solution is then taken using a pipette and the remaining dye is washed using running water four times and then dried at room temperature. A total of 200 μL of 95% ethanol was added to each well to fix the color bound to the biofilm cells. The microplate is closed and allowed to stand for 30 minutes and then an optical density (OD) reading is taken at a wavelength of 570 nm using a microplate reader (Bio Tech Epoch Microplate Spectrophotometer).

### Methylthiazol Tetrazolium Assay

Furthermore, 10 μL of methylthiazol tetrazolium (MTT; Invitrogen by Thermo Fisher Scientific) was added dropwise into each well. The microplate is re-incubated for 3 to 4 hours at 37°C in the incubator. After that, 50 μL dimethyl sulfoxide (Vivantis – ACS Grade) is added to each *well* and vibrated with a microplate shaker for 5 minutes until the formazan crystals are dissolved. The OD value of the formazan is read with a microplate reader (Bio Tech Epoch Microplate Spectrophotometer) at 540 nm.

### Data Analysis for Crystal Violet Assay

The OD value that has been obtained through reading with Elisa Reader at a wavelength of 570 nm is calculated through the following formula:





Based on the results obtained from the above formula, the interpretation of OD *isolate* values is grouped into four groups as follows:









### Data Analysis for *Methylthiazol Tetrazolium* Assay

The OD value obtained through reading with Elisa Reader at a wavelength of 540 nm is calculated through the following formula:



Based on these formulas, the resulting data will be categorized into three groups of interpretation results, namely:







## Result

### Optical Density Results on Crystal Violet Assay

In the crystal violet assay, the absorbance results show the ability to form biofilms. Table 1 shows that control treatments fall under the category of NBF, while other treatments fall under the category of HBF.

**Table 1:** Optical density (OD) value in crystal violet assay

Absorbance	Control	*S. mutans*	*L. plantarum*	*C. albicans*	*S. mutans* + *L. plantarum*	*S. mutans* + *C. albicans*	*S. mutans* + *L. plantarum* + *C. albicans*
**Mean**	0.082	2.241	2.018	2.992	2.028	2.887	2.994
**OD value**	−0.067	1.991	1.869	2.842	1.879	2.738	2.844
**Interpretation**	NBF	HBF	HBF	HBF	HBF	HBF	HBF

Abbreviations: HBF, high biofilm forming; NBF, no biofilm forming; OD, optical density.

### Optical Density Results on MTT Assay

In the MTT assay, the absorbance results show the metabolic activity of bacteria. Table 2 shows that the control treatment is included in the category of LCP. Monospecies biofilms *S. mutans* and *L. plantarum* fall into the NCP category. Meanwhile, dual-biofilms formed by *S. mutans* together with either *L. plantarum* or *C. albicans* and polymicrobial biofilms formed by these three bacteria fall into the category of ICP.

**Table 2:** Optical density (OD) value in MTT assay

Absorbance	Control	*S. mutans*	*L. plantarum*	*C. albicans*	*S. mutans* + *L. plantarum*	*S. mutans* + *C. albicans*	*S. mutans* + *L. plantarum* + *C. albicans*
**Mean**	0.052	1.15	1.019	1.375	1.383	1.373	1.858
**OD value**	0	1.098	0.968	1.323	1.33	1.32	1.806
**Interpretation**	LCP	NCP	NCP	ICP	ICP	ICP	ICP

Abbreviations: ICP, increased cell proliferation; LCP, low cell proliferation; OD, optical density.

### Discussion

Biofilm is a layer that is tightly attached to a surface where it consists of colonies of microorganism cells. 11 In this analysis, was carried out to see the ability of biofilm formation from *S. mutans, L. plantarum,* and *C. albicans* bacteria, both monospecies biofilms and multispecies biofilms through crystal violet assay indicated by OD values and MTT assay to see the metabolic activity of treatment bacteria. Crystal violet assay and MTT assay cannot show specifically what gene expression is working, but only show biofilm formation and metabolic activity that is seen as a result of the gene expression that is working.

*Streptococcus mutans* produces one of the inducers called CSP to carry out the quorum sensing process. This CSP regulates the two-component signal transduction system and genetic competence. The two-component signal transduction system involves a sensor-regulator system encoded by *comD* and *comE* . 12 CSP is encoded as a precursor by the *ComC* gene, which is useful for regulating gene expression. CSP will later bind to histidine kinase receptors encoded by the *ComD* gene when the amount has reached a threshold and accumulated in the environment. CSP binding with histidine kinase receptors is useful for regulating communication signal responses which will later trigger the process of autophosphorylation and activation of the response regulator encoded by the *ComE* gene. This activation will cause message transduction that triggers the activation of target genes used in biofilm formation, bacteriocin production, and stress response. 13 14 15 In the formation of biofilms, *S. mutans* produces three Gtf enzymes, namely, GtfB, GtfC, and GtfD, with each encoded by the *gtfB* , *gtfC* , and *gtfD* genes. 16

In the crystal violet assay, in addition to controls, all biofilms formed fall into the HBF category where all treatments have OD values four times greater than OD cut-off values. Meanwhile, in the MTT assay, the control is included in LCP. *Streptococcus mutans* and *L. plantarum* are included in the NCP category, while other treatments are included in the ICP category.

Monospecies biofilm formation capabilities of *S. mutans* show higher results compared to dual-biofilm of *S. mutans* with *L. plantarum* . This can be possible due to the inhibition of biofilm of *S. mutans* through mechanisms of quorum sensing. The inducer from *L. plantarum* , known as AI-2, has the ability to cause EPS degradation, thus resulting in a reduction in the biofilm formation of *S. mutans* . 17 This is in line with the findings of Srivastava et al *,* which shows that *L. plantarum* can significantly decrease the production of *gtfB, gtfC,* and *gtfD* from *S. mutans* . As a result, attachment of *S. mutans* decreased, which has an effect on decreasing biofilm formation. 18 However, these results are not in line with the results in the MTT assay, which may occur due to the role of *S. mutans* , which can increase metabolic activity of *L. plantarum* by creating an environment rich in sugar. Some possibilities that can cause the growth of biofilms with low levels of metabolic activity, such as the presence of quorum sensing that regulates biofilm formation, can reset metabolic activity collectively, which can possibly lead to a decrease in metabolic activity. In addition, the variability of microorganisms in biofilms has different levels of metabolic activity in each microorganism, where some microorganisms may have high levels of metabolic activity, while others low metabolic activity. 3

The biofilm formation ability and metabolic activity of *S. mutans* monospecies biofilms compared to dual-biofilms of *S. mutans* with *C. albicans* showed lower OD values. The possibility is the presence of quorum sensing molecules from *C. albicans* that can increase the formation of *S. mutans* biofilms, namely farnesol. This farnesol can lead to increased GtfB production leading to increased biofilm formation. 19 20 Actually, *C. albicans* has another inducer named tyrosol which also has an effect on the biofilm of *S. mutans* . High tyrosol concentrations can lead to decreased *S. mutans* biofilms, which may occur through cell membrane integrity associated with the loss of K ^+^ ions. However, between the two, farnesol has a more dominant effect, so the effects of farnesol are more dominant. 21

Polymicrobial biofilms formed by the three bacteria had the highest biofilm growth ability and metabolic activity of all treatments. This is because of the process of biofilm formation, where at the stage of microcolony formation, bacteria will multiply and aggregate. Then the multi-layered cells will accumulate through proliferation and EPS will be produced. Thus, if metabolic activity is represented by a high proliferation rate, the growth of biofilms is also high. Conversely, if the proliferation rate is low, the growth of biofilms is also low. 22

Biofilm formation ability and metabolic activity in monospecies biofilms of *C. albicans* showed higher value than dual-biofilms of *C. albicans* with *S. mutans* . This is likely to happen because according to Zago et al, who conducted a dual-biofilm study of *C. albicans* with bacteria, between *C. albicans* yeast cells and these bacteria there will be an antagonistic interaction so that the biofilms formed and metabolic activity that occurs decrease. 23

## Conclusion

*Lactobacillus plantarum* is capable of inhibiting the formation of *S. mutans* biofilms. Meanwhile, *C. albicans* can increase the formation of *S. mutans* biofilms so it can accelerate the caries process. Further research on both inducers is suggested that is useful for alternative references to caries preventive treatments in the future.
